# Quercetin and Isorhamnetin Attenuate Benzo[a]pyrene-Induced Toxicity by Modulating Detoxification Enzymes through the AhR and NRF2 Signaling Pathways

**DOI:** 10.3390/antiox10050787

**Published:** 2021-05-16

**Authors:** Min Kim, Seung-Cheol Jee, Kyeong-Seok Kim, Hyung-Sik Kim, Kyoung-Nae Yu, Jung-Suk Sung

**Affiliations:** 1 Department of Life Science, Dongguk University-Seoul, Biomedi Campus, 32 Dongguk-ro, Ilsandong-gu, Goyang 10326, Gyeonggi-do, Korea; pipikimmin@naver.com (M.K.); markjee@naver.com (S.-C.J.); ryoukr@naver.com (K.-N.Y.); 2 Division of Toxicology, School of Pharmacy, Sungkyunkwan University-Suwon, Suwon 16419, Gyeonggi-do, Korea; caion123@nate.com (K.-S.K.); hkims@skku.edu (H.-S.K.)

**Keywords:** benzo[a]pyrene, quercetin, isorhamnetin, xenobiotic metabolism, AhR, NRF2

## Abstract

Benzo[a]pyrene, classified as a Group 1 carcinogen, is metabolized to B[a]P-7,8-dihydrodiol-9,10-epoxide (BPDE), causing DNA mutations and eventually cancer. Quercetin is a dietary flavonoid abundant in fruits and vegetables. After quercetin intake, quercetin’s metabolites isorhamnetin and miquelianin are more highly concentrated than quercetin in the human plasma. In this study, we investigated the molecular mechanisms associated with the cytoprotective effect of quercetin and its metabolites against benzo[a]pyrene from a detoxification perspective. Quercetin and its metabolite isorhamnetin reduced benzo[a]pyrene-induced cytotoxicity, whereas the metabolite miquelianin did not mitigate benzo[a]pyrene-induced cytotoxicity. Moreover, quercetin and isorhamnetin reduced intracellular levels of BPDE-DNA adducts. The formation and elimination of BPDE is mediated by the xenobiotic detoxification process. Quercetin and isorhamnetin increased the gene and protein expression levels of phase I, II, and III enzymes involved in xenobiotic detoxification. Furthermore, quercetin and isorhamnetin induced the translocation of aryl hydrocarbon receptor (AhR) and nuclear factor erythroid 2-related factor 2 (NRF2), which regulate the expression level of phase enzymes. Our results suggest that quercetin and isorhamnetin promote the metabolism, detoxification, and elimination of B[a]P, thereby increasing anti-genotoxic effects and protecting against B[a]P-induced cytotoxicity.

## 1. Introduction

Benzo[a]pyrene (B[a]P), a polycyclic aromatic hydrocarbon, is produced from the incomplete combustion of organic materials [1]. It is a ubiquitous compound found in cigarette smoke, burning coal smoke, and various foods, especially fire-grilled red meats [2]. Previous studies have shown that 97% of humans’ total daily B[a]P exposure occurs through food [3,4]. B[a]P is well known to be a pro-carcinogen and is classified as a Group 1 carcinogen to humans [5,6,7]. Once B[a]P is absorbed into the intestinal tract, it is metabolized in the liver and transformed into various metabolites by cytochrome P450 (CYP) enzymes. Generally, B[a]P is sequentially oxidized to B[a]P-7,8-epoxide, B[a]P-7,8-dihydrodiol, and B[a]P-7,8-dihydrodiol-9,10-epoxide (BPDE). The metabolites of B[a]P are mutagenic and highly carcinogenic, especially BPDE [8]. As the ultimate metabolite of B[a]P, BPDE intercalates into the DNA, covalently bonding to the nucleophilic guanine bases, which causes mutations and eventually cancer [1]. Xenobiotic detoxification of BPDE converts reactive metabolites into less reactive compounds and is necessary to prevent BPDE’s mutagenic and carcinogenic actions [9].

Xenobiotic detoxification metabolism consists of three phases. The phase I enzymes such as cytochrome P450 family 1 subfamily A member 1 (CYP1A1) and cytochrome P450 family 1 subfamily B member 1 (CYP1B1) introduce oxygen molecules to xenobiotic compounds [10]. The converted compounds are then conjugated to glutathione (GSH), sulfate, or glucuronic acid by phase II enzymes such as glutathione S-transferase alpha 1 (GSTA1) and glutathione S-transferase pi 1 (GSTP1). The reactive compounds are usually stabilized in phase II [11]. Finally, in phase III, the conjugated xenobiotic compounds are pumped out and eliminated from the cells by efflux transporters such as ATP-binding cassette subfamily C member 2 (ABCC2) and ATP-binding cassette subfamily C member 3 (ABCC3) [12]. The aryl hydrocarbon receptor (AhR), a ligand-activated transcription factor, is activated by B[a]P and induces the expression of genes involved in xenobiotic metabolism, such as CYP1A1 and CYP1B1 [13]. Nuclear factor erythroid 2-related factor 2 (NRF2) is activated not by ligands, as is AhR, but by endogenous compounds that generate a redox signal. A previous study showed that the expression of phase II and III genes, such as GSTA1, GSTP1, ABCC2, and ABCC3, is dependent on NRF2 [14]. Interestingly, both AhR and NRF2 can be activated by cross-talk between each other [15].

Quercetin, the most abundant flavonoid in plant-based foods such as apples (58.2 mg/100 g dry weight), onions (826 mg/100 g dry weight), and red wine (47.3 mg/100 g dry weight), has been studied extensively for its antioxidant properties [16]. Once absorbed into the body, quercetin is converted to isorhamnetin by catechol-*O*-methyltransferase (COMT) in the liver via methylation at the 3′ position of the B-ring. After intake, quercetin’s metabolites such as isorhamnetin and miquelianin have a higher concentration in the human plasma than does quercetin itself [17]. Numerous studies have focused on quercetin’s protective effect against B[a]P-induced carcinogenesis [18]; however, the overall mechanism of detoxification by quercetin and isorhamnetin has not been investigated in detail [19]. 

In this study, we aimed to investigate the detoxification mechanisms and effects of quercetin and its metabolite isorhamnetin. Previous studies have focused primarily on the antioxidant efficacy of quercetin in reducing oxidative stress; however, the specific detoxification mechanisms remain unclear. Furthermore, no studies have been completed on the detoxification mechanisms of isorhamnetin against B[a]P-induced toxicity. Here, we focused on xenobiotic detoxification and the signaling pathways that regulate phase I, II, and III enzymes involved in xenobiotic detoxification.

## 2. Materials and Methods 

### 2.1. Chemicals and Reagents

Quercetin, isorhamnetin, miquelianin, B[a]P, 4,6-diamidino-2-phenylindole dihydrochloride (DAPI), nuclease P1, dimethyl sulfoxide (DMSO), and Triton X-100 were purchased from Sigma-Aldrich (St. Louis, MO, USA). Minimum essential medium (MEM), penicillin/streptomycin, fetal bovine serum (FBS), sodium pyruvate, and trypsin-EDTA were purchased from Welgene (Daegu, South Korea). Phosphate-buffered saline (PBS) was purchased from Biosesang (Seongnam, South Korea). Fluorescent mounting medium was purchased from Dako (Santa Clara, CA, USA). Antibodies (anti-NRF2, AhR, CYP1A1, GSTA1, CYP1A1, CYP1B1, ABCC2, ABCC3, and β-actin) and Alexa 488-conjugated anti-rabbit secondary antibodies were purchased from Cell Signaling Technology (Danvers, MA, USA).

### 2.2. Animals and Housing

Thirty 5-week-old male Sprague-Dawley (SD) rats were obtained from Orient Bio (Seongnam, South Korea), initially weighing around 120 g. All animals were maintained under a specific-pathogen-free (SPF) condition room with a 12-h light/dark cycle. They were housed at ambient air temperature (23 ± 2 °C) under humidity (55%) and fed ad libitum with distilled water and rodent chow. The animals were randomly divided into three groups (*n* = 5~6): (1) a control group, in which the rats received a daily oral administration of corn oil; (2) a B[a]P-treated group, in which the rats received a daily oral administration of B[a]P (20 mg/kg) dissolved in corn oil; and (3) a B[a]P + quercetin-treated group, in which the rats received a daily oral administration of quercetin (2.5, 7.5, and 15 mg/kg) with B[a]P dissolved in corn oil. Each group was fed its respective treatment for 30 days. The animals were sacrificed 30 days after oral administration. The harvested liver and stomach were immediately frozen using liquid nitrogen and stored at −80 °C until use for further experiments. DNA was extracted from 20–40 mg of each tissue and used for BPDE-DNA adduct and 8-oxo-dG assay. The study protocol was approved by Sungkyunkwan University Laboratory Animal Care Service (SKKU-2013-000105, 23 March 2013) in accordance with the Ministry of Food and Drug Safety (MFDS) Animal Protection of Korea (Oh-Song, Korea). All experimental were performed following Guide for the Care and Use of Laboratory Animals.

### 2.3. Cell Culture and Treatment

A HepG2 cell line was purchased from the American Type Culture Collection (VA, USA). The HepG2s were cultured with MEM supplemented with 10% FBS, 100 U/mL penicillin and streptomycin, and 1 mM sodium pyruvate in a 150-mm^2^ cell culture dish. All cells were incubated at 37 °C in 5% CO₂, and the medium was changed every other day. To determine the effects of flavonoid compounds, 3 × 10^4^ cells/cm^2^ HepG2 cells were seeded and cultured in MEM with B[a]P at final concentration of 1.0 to 40 μM for 48 h, with or without the flavonoid compounds. The cells were then used for further analysis or extraction.

### 2.4. Cell Viability Analysis

Cell viability was analyzed using the EZ-CYTOX reagent (DOGEN, Daejeon, South Korea). The cells were seeded into 96-well plates at 1 × 10^4^ cells/well. Next, B[a]P, quercetin, isorhamnetin, or miquelianin was added at concentrations of 0, 1, 5, 10, 20, and 40 μM. After treatment with each compound for 48 h, the cell culture medium containing the compounds was changed to a medium containing the EZ-CYTOX reagent. The cultures were then incubated for 2 h and the absorbance was measured at 450 nm using a microplate reader (VersaMax™, Molecular Devices, CA, USA). The cell viabilities of the flavonoid-treated groups were compared with those of the untreated control group.

### 2.5. Quantification of 8-Hydroxydeoxyguanosine (8-oxo-dG) 

An enzyme-linked immunosorbent assay (ELISA) kit for 8-oxo-dG was purchased from Cell Biolabs (CA, USA). The assay was performed according to the manufacturer’s protocol. In brief, DNA was isolated, incubated at 95 °C for 5 min. The DNA sample was digested to nucleosides by incubating with 10 units of nuclease P1 for 2 h at 37 °C. Then, 10 units of alkaline phosphatase (Takara, Japan) and 100 mM Tris buffer (pH 7.5) was added and incubated for 15 min at 37 and 50 °C, respectively. The reaction mixture was 5× *g* for 5 min at 6000× *g*, and the supernatant was used for the assay. The enzyme reaction was stopped by adding 100 µl of stop solution, and the absorbance was measured using a microplate reader at 450 nm. 

### 2.6. BPDE-DNA Adduct Formation Analysis

We determined the formation of BPDE-DNA adducts using ELISA according to the manufacturer’s protocol. DNA was extracted using a QIAamp DNA Mini Kit (Qiagen, Germantown, MD, USA). For this assay, DNA samples were diluted to 2 µg/mL using PBS. The reaction was stopped using a stop solution, and the absorbance was measured using a microplate reader at 450 nm.

### 2.7. Intracellular Metabolite Extraction and HPLC Analysis

Treated cells were dissolved in ethyl acetate following the addition of each compound. The dissolved cells were then vaporized and analyzed using an Agilent 1100 Series HPLC system (Hewlett-Packard, Palo Alto, CA, USA). Chromatography was conducted using a 5-µm Kinetex C18 column (4.6 mm × 250 mm, Phenomenex, Torrance, CA, USA) at 25 °C, with a wavelength of 254 nmol and a flow rate of 1.0 mL/min. HPLC separation was carried out using the following gradient: 25–30 min for 100% acetonitrile in 0.1% acetic acid, and 0–40 min for 50% acetonitrile in 0.1% acetic acid. The B[a]P, B[a]P-7,8-dihydrodiol, and BPDE standards were purchased from the MRIGlobal Chemical Carcinogen Repository (Kansas, MO, USA).

### 2.8. Quantitative Reverse Transcription-Polymerase Chain Reaction (qRT-PCR)

Total RNA was acquired using a TRIzol reagent (Life Technologies, Carlsbad, CA, USA). cDNA was synthesized from 2 μg of the total RNA and used for qRT-PCR (CFX Connect™ Real-Time PCR Detection System; Bio-Rad, Hercules, CA, USA) with SYBR Green PCR Master Mix (KAPA, Wilmington, MA, USA). Amplification was set with three-step cycling: initial denaturation at 95 °C for 3 min, 50 cycles of denaturation at 95 °C for 10 s, annealing at 60 °C for 10 s, and extension at 72 °C for 10 s.

### 2.9. Western Blot Analysis

Total cell lysates were extracted using a radioimmunoprecipitation assay (RIPA) buffer (Biosolution, Seoul, South Korea) containing protease inhibitor cocktail, phosphatase inhibitor cocktail 2, and phosphatase inhibitor cocktail 3 (Sigma-Aldrich, St. Louis, MO, USA). A 30-µg aliquot of cellular proteins was separated using 10% SDS-polyacrylamide gel electrophoresis and transferred to a polyvinylidene difluoride (PVDF) membrane (Millipore, Burlington, MA, USA). The membrane was incubated with primary and secondary antibodies, and ECL Plus Western blotting detection reagent (Amersham Bioscience, Buckinghamshire, UK) was added to the membrane. β-actin was used as a loading control. Images were obtained using the Bio-Rad ChemiDoc XRS System (Hercules, CA, USA) and analyzed using Quantity One imaging software (Bio-Rad Laboratories, Hercules, CA, USA).

### 2.10. Immunofluorescence Analysis

Following treatment with the flavonoid compounds, the cells were fixed with 4% formaldehyde for 15 min and then treated with 0.25% Triton X-100. Next, the cells were blocked with 1% bovine serum albumin (Sigma-Aldrich, St. Louis, MO, USA) and sequentially incubated with primary and secondary antibodies for 1 h each. After incubation, the cells were stained using DAPI and mounted using mounting medium. The images were acquired by confocal microscopy (Olympus, Tokyo, Japan) and analyzed using ImageJ software (National Institutes of Health, Bethesda, MD, USA).

### 2.11. Statistical Analysis

All data are expressed as the mean ± SEM of at least three independent experiments. Statistical analysis was performed by one-way ANOVA with Tukey’s multiple comparison analysis. A *p* value of less than 0.05 was considered statistically significant.

## 3. Results

### 3.1. Cytoprotective Effect of Quercetin and Its Metabolites Against B[A]P-Induced Toxicity

**Figure 1:**
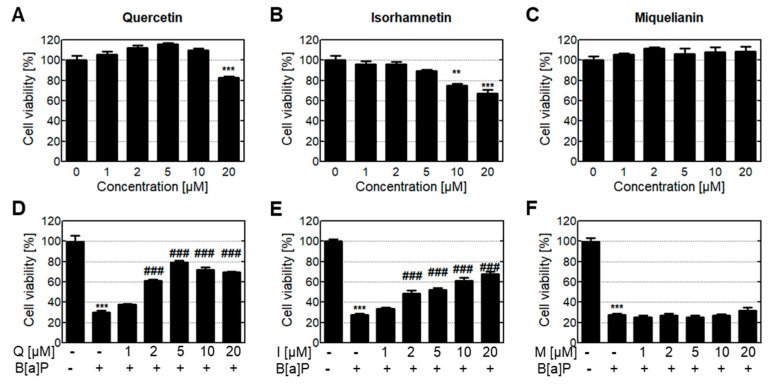
Cytotoxic effect of quercetin and its metabolites on HepG2 cells treated with or without B[a]P. The cytotoxicity of the compounds (**A**) quercetin, (**B**) isorhamnetin, and (**C**) miquelianin was determined. Then, the protective effect of each compound against B[a]P was evaluated. Cells were treated with a combination of 10 μM B[a]P and (**D**) quercetin, (**E**) isorhamnetin, or (**F**) miquelianin. ** *p* < 0.01, *** *p* < 0.001 in comparison with untreated cells; ### *p* < 0.001 in comparison with B[a]P-treated cells.

First, we analyzed the cytotoxicity of quercetin and its metabolites. HepG2 cells are a type of liver cell used in drug metabolism studies due to their property of drug-induced metabolic enzyme expression. Low quercetin and isorhamnetin concentration resulted in no significant cytotoxicity (Figure 1A,B); however, cells in the group treated with 20 μM quercetin had 83% viability. Cells in the groups treated with 10 and 20 μM of isorhamnetin had 80% and 70% viability, respectively. These results showed that high quercetin and isorhamnetin concentrations were toxic to HepG2 cells, whereas miquelianin was not toxic to these cells (Figure 1A–C). To investigate the protective effect of quercetin and its metabolites on B[a]P-induced cytotoxicity, HepG2 cells exposed to quercetin and its metabolites were subsequently treated with B[a]P (Figure 1D–F). We found that cells treated with both quercetin and B[a]P recovered to their normal viability, and quercetin with B[a]P increased cell viability up to 5 μM in a dose-dependent relationship. 

The degree of cell viability recovery after treatment with quercetin concentrations of 10 and 20 μM with B[a]P appeared to be slightly reduced due to self-toxicity caused by the high concentration of quercetin. Treatment with isorhamnetin increased the cell viability in a dose-dependent manner, although high concentrations of isorhamnetin are cytotoxic to HepG2 cells. Cells treated with miquelianin had no significant change in B[a]P-induced cytotoxicity. In further experiments, quercetin and isorhamnetin were treated at 5 μM; at this concentration, none of the treatments showed evidence of cytotoxicity, and B[a]P-induced toxicity was reduced.

### 3.2. Anti-genotoxic Effects Of Quercetin and Isorhamnetin via The Reduction of Intracellular B[A]P and Metabolites 

**Figure 2:**
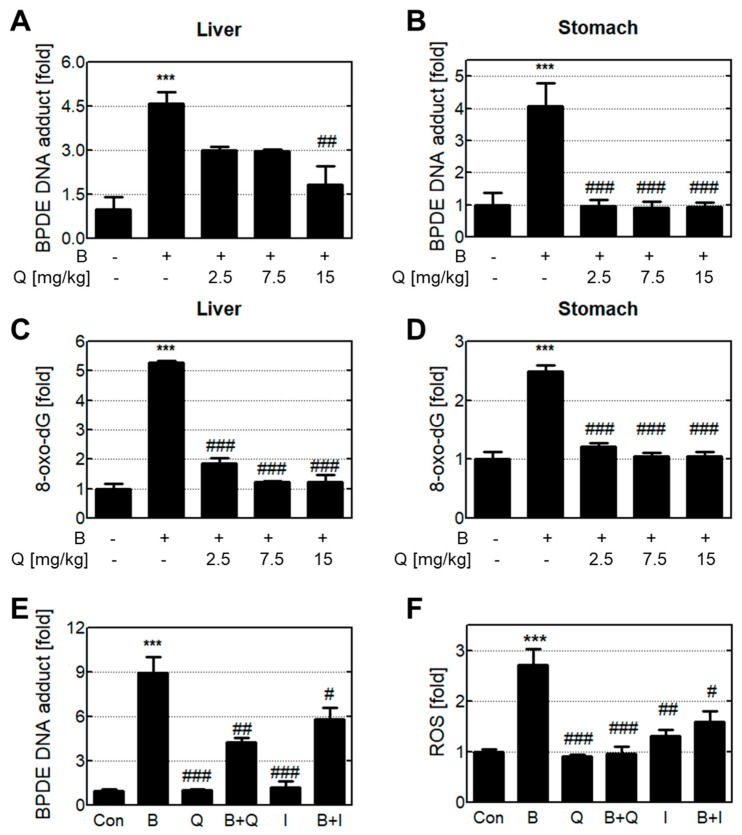
Quercetin and isorhamnetin reduce reactive oxygen species (ROS) and BPDE-DNA adduct formation caused by B[a]P. Formation of BPDE-DNA adduct in the (**A**) liver and (**B**) stomach was analyzed after orally administering quercetin for 30 days. Formation of 8-oxo-dG in the (**C**) liver and (**D**) stomach was also analyzed. Intracellular levels of (**E**) BPDE-DNA adduct and (**F**) ROS were determined after treatment with quercetin for 48 h. *** *p* < 0.001 compared with the control; # *p* < 0.05, ## *p* < 0.01, ### *p* < 0.001 compared with B[a]P-treated cells. Con: control; B: benzo[a]pyrene (B[a]P) (10 μM); Q: quercetin (5 μM); I: isorhamnetin (5 μM).

To observe the reduction effect of quercetin on DNA damage such as BPDE-DNA adduct and 8-oxo-dG in the stomach and liver tissues, various concentrations of quercetin with B[a]P were orally administered to rat (Figure 2A–D). In the groups treated with B[a]P, the BPDE-DNA adducts in the stomach and liver tissues increased more than 4-fold over the untreated group. However, the group that was administered quercetin co-treated with B[a]P had significantly decreased BPDE-DNA adducts compared with the group that was administered B[a]P only. The concentration of 8-oxo-dG was also increased in both the stomach and liver tissues after B[a]P administration. As mentioned above, since quercetin is mainly metabolized to isorhamnetin after ingestion, we analyzed the reduction effect of quercetin and isorhamnetin on BPDE-DNA adduct and 8-oxo-dG in human liver cells (Figure 2E,F). The cells treated with B[a]P only had much higher levels of BPDE-DNA adduct and 8-oxo-dG compared with the untreated group. The groups treated with either quercetin or isorhamnetin in combination with B[a]P had reduced levels of both BPDE-DNA adduct and 8-oxo-dG. 

**Figure 3:**
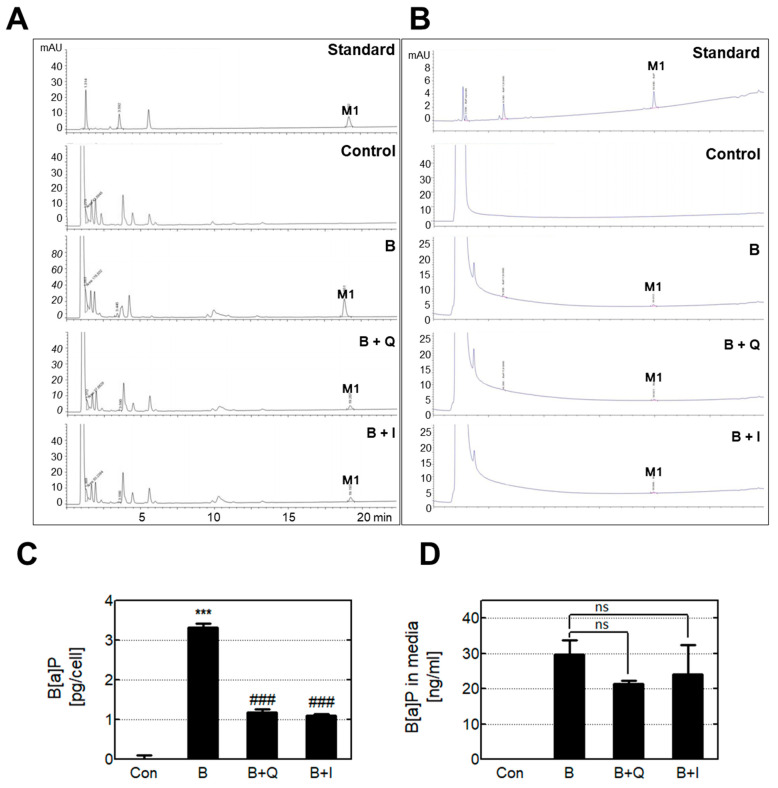
Reduction of intracellular B[a]P by quercetin and isorhamnetin. (**A**) Intracellular B[a]P and (**B**) remaining B[a]P in the cell culture media were analyzed by HPLC. These data were quantified and are presented graphically for (**C**) Intracellular B[a]P and (**D**) remaining B[a]P in cell culture media. *** *p* < 0.001 compared with the control; ### *p* < 0.001 compared with B[a]P-treated cells; ns: not significant. M1: benzo[a]pyrene (B[a]P); Con: control; B: benzo[a]pyrene (B[a]P) (10 μM); Q: quercetin (5 μM); I: isorhamnetin (5 μM).

To measure intracellular B[a]P, cells were lysed in ethyl acetate, and then B[a]P in the whole extract was analyzed by HPLC (Figure 3A,C). B[a]P was not detected in the untreated cell extract, whereas it was detected at high levels in B[a]P-treated cell extracts. In cells treated with either quercetin or isorhamnetin in combination with B[a]P, the intracellular B[a]P was decreased compared with cells that had been treated with B[a]P only. To examine whether cellular absorption of B[a]P was affected by quercetin or isorhamnetin, the amounts of both the intracellular B[a]P and the B[a]P remaining in the cell culture media were analyzed using HPLC (Figure 3B,D). The amount of remaining B[a]P in the cell culture media did not differ significantly between cells treated with B[a]P only and those co-treated with B[a]P and quercetin or isorhamnetin. These results indicate that quercetin and isorhamnetin reduce the intracellular B[a]P level, but do not affect B[a]P’s cellular absorption rate. 

### 3.3. Effects of Quercetin and Isorhamnetin on Phase Enzyme Expression

**Figure 4:**
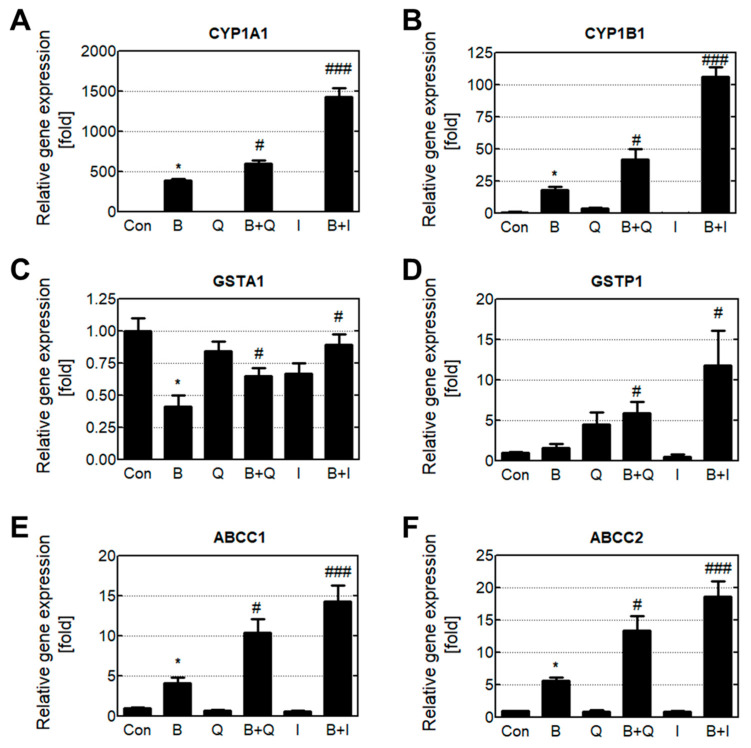
Increased gene expression levels of B[a]P metabolic enzymes induced by quercetin and isorhamnetin. B[a]P was metabolized by phase enzymes. The gene expression levels of the phase I enzymes (**A**) *CYP1A1* and (**B**) *CYP1B1*, the phase II enzymes (**C**) *GSTA1* and (**D**) *GSTP1*, and the phase III enzymes (**E**) *ABCC2* and (**F**) *ABCC3* were analyzed. * *p* < 0.05 compared with untreated cells; # *p* < 0.05, ### *p* < 0.001 compared with B[a]P-treated cells. Con: control; B: benzo[a]pyrene (B[a]P) (10 μM); Q: quercetin (5 μM); I: isorhamnetin (5 μM).

The gene expression levels of detoxification phase I, II, and III enzymes were analyzed using real-time quantitative PCR (Figure 4). Cells treated with B[a]P only had increased expression of the phase I enzymes *CYP1A1* and *CYP1B1* compared with cells in the untreated group. In HepG2 cells, CYP enzymes exhibited drug-dependent expression and were rarely expressed in the untreated cells. Treating cells with quercetin or isorhamnetin in combination with B[a]P significantly increased *CYP1A1* and *CYP1B1* expression. Cells treated with B[a]P only had decreased expression of the phase II enzyme *GSTA1*, but increased expression of *GSTP1*. Treatment with quercetin and isorhamnetin resulted in recovered *GSTA1* levels, and significantly increased *GSTP1* expression compared with B[a]P alone. Additionally, treating cells with quercetin and isorhamnetin increased the expression level of the phase III enzymes *ABCC1* and *ABCC2* more than 2-fold compared with B[a]P alone. 

**Figure 5:**
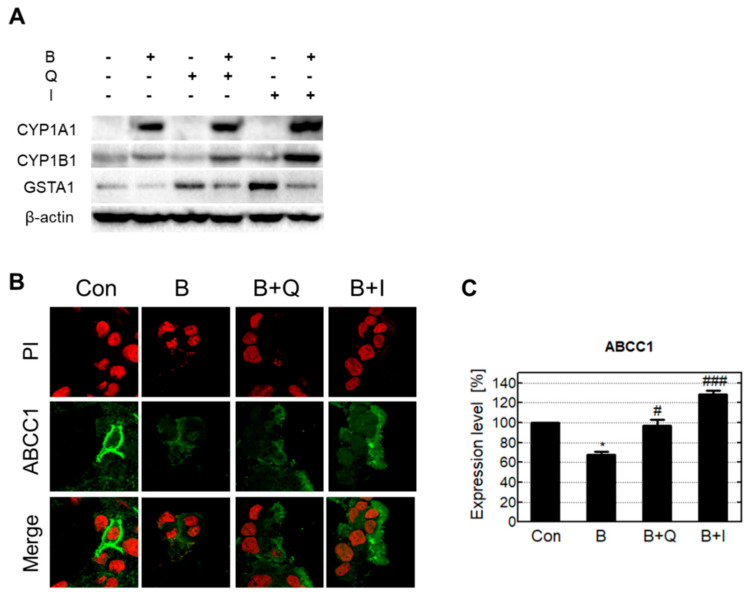
Increased protein expression levels of B[a]P metabolic enzymes induced by quercetin and isorhamnetin. (**A**) Analysis of intracellular protein levels of the phase I enzymes CYP1A1 and CYP1B1 and the phase II enzyme GSTA1. (**B**) Analysis of intramembrane protein levels of ABCC1. (**C**) Quantification and graphical presentation of ABCC1 expression levels. Con: control; B: benzo[a]pyrene (B[a]P) (10 μM); Q: quercetin (5 μM); I: isorhamnetin (5 μM). * *p* < 0.05, # *p* < 0.01, ### *p* < 0.001.

The intracellular protein levels of the phase I and II enzymes were analyzed by Western blotting. Since the phase III enzymes (transporter proteins) are expressed in the cell membrane, their expression levels were analyzed using immunocytochemistry (Figure 5). Similarly to the phase enzymes’ gene expression levels, cells treated with B[a]P only had increased CYP1A1 and CYP1B1 protein levels compared with the control group. Cells co-treated with either quercetin or isorhamnetin in combination with B[a]P expressed more CYP enzymes compared with cells that were treated with B[a]P only. Cells treated with B[a]P only had decreased GSTA1 protein levels, whereas cells co-treated with either quercetin or isorhamnetin in combination with B[a]P had recovered GSTA1 protein levels. Cells treated with quercetin and isorhamnetin had higher ABCC1 enzyme expression compared with cells that were treated with B[a]P only. These results show that quercetin and isorhamnetin co-treated with B[a]P increase both the gene expression and protein levels of phase I, II, and III enzymes compared with B[a]P alone. 

### 3.4. Effect of Quercetin and Isorhamnetin on Ahr and NRF2 Translocation

**Figure 6:**
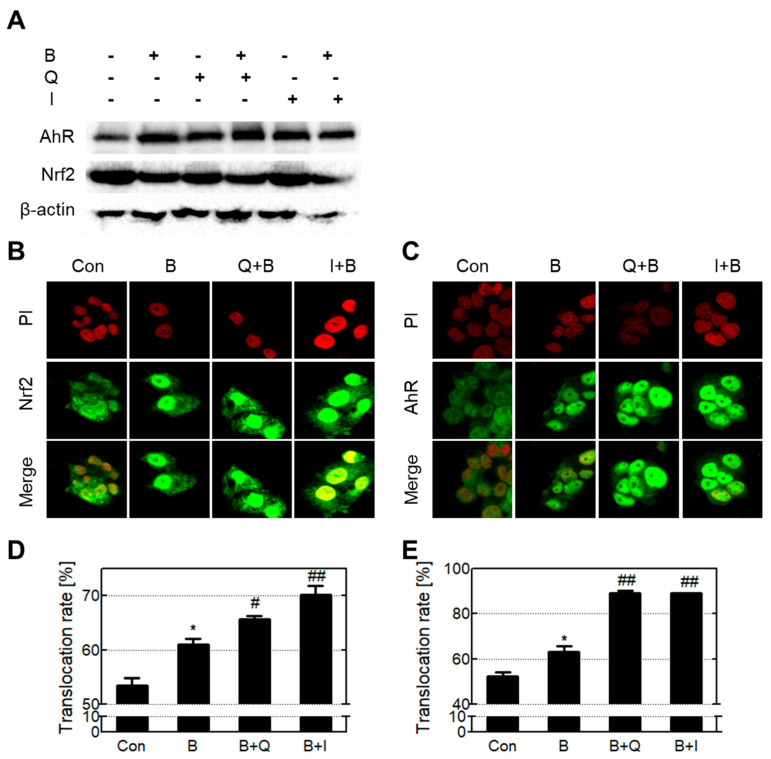
Increased translocation of NRF2 and AhR induced by quercetin and isorhamnetin. (**A**) Examination of intracellular protein levels of NRF2 and AhR. (**B**,**C**) Analysis of (**B**) NRF2 and (**C**) AhR translocation by confocal microscopy. (**D**,**E**) Quantification and graphical presentation of the translocation rates of (**D**) NRF2 and (**E**) AhR. * *p* < 0.05 compared with the control; # *p* < 0.05, ## *p* < 0.01, compared with B[a]P-treated cells. Con: control; B: benzo[a]pyrene (B[a]P) (10 μM); Q: quercetin (5 μM); I: isorhamnetin (5 μM).

The transcription factors AhR and NRF2 regulate phase enzyme expression levels (Figure 6A). None of the experimental treatment groups experienced significant changes in AhR and NRF2 protein levels compared with the control. When activated, AhR and NRF2 translocate to the nucleus and bind with specific DNA sequences that regulate phase enzymes. Therefore, we analyzed the translocation of AhR and NRF2 by fluorescently staining the intracellular and intranuclear proteins of AhR and NRF2 using immunocytochemistry (Figure 6B–E). Cells treated with B[a]P only showed increased translocation of both AhR and NRF2 compared with the control group, and cells co-treated with either quercetin or isorhamnetin in combination with B[a]P showed a further increase in translocation of AhR and NRF2 compared with cells that were treated with B[a]P only. These results indicate that quercetin and isorhamnetin increase the translocation of AhR and NRF2 without affecting their expression levels.

## 4. Discussion

B[a]P, a well-known Group 1 carcinogen, is exposed to the body through fire-cooked foods, especially grilled meat. Quercetin is an important dietary flavonoid that is abundant in plant-based foods such as apples, onions, and red wine, and which has been studied extensively for its antioxidant properties [20]. After consuming quercetin-containing foods, quercetin is conjugated with glucuronic acid, sulfate, or methyl groups in the human plasma [21]. In a previous study, the three major compounds identified from quercetin in plasma were 3-methylquercetin (isorhamnetin), quercetin 3-*O*-glucuronide (miquelianin), and quercetin; in the same study, plasma levels of isorhamnetin were 4 times higher than the plasma level of quercetin [22]. In the present study, quercetin and its metabolites (isorhamnetin and miquelianin) were investigated for their reversal of B[a]P-induced toxicity.

We first analyzed the cytotoxic effects of quercetin and its metabolites on HepG2 liver cells. Quercetin and isorhamnetin were cytotoxic at high concentrations, whereas miquelianin was not toxic at the same concentration (Figure 1A–C). It is possible that conjugation with glucuronides in phase II xenobiotic metabolism contributes to miquelianin’s low toxicity. Quercetin and isorhamnetin had a cytoprotective effect against B[a]P-induced toxicity, whereas miquelianin had no such effect (Figure 1D–F). Quercetin, isorhamnetin, and miquelianin have all been reported to have antioxidant properties [23]; in particular, isorhamnetin and miquelianin were reported to be twice as effective as quercetin [22]. In our experiments, quercetin and isorhamnetin treated at 5 μM showed no cytotoxicity in a single treatment and had a mitigating effect on B[a]P-induced toxicity.

B[a]P metabolism, which occurs mainly in the liver, stimulates reactive oxygen species (ROS) production, resulting in oxidative DNA damage such as 8-oxo-dG formation [24]. Furthermore, BPDE, a metabolite of B[a]P, has an epoxide ring structure that has a strong tendency to covalently bind with DNA [25]. These 8-oxo-dG and BPDE-DNA adducts can induce the distortion of the double-helical DNA structure, potentially causing cancer to develop [26]. Therefore, we examined the presence of 8-oxo-dG and BPDE-DNA adducts in tissues after oral administration of quercetin with or without B[a]P for 30 days. Our results showed that quercetin intake inhibited the formation of both 8-oxo-dG and BPDE-DNA adducts in the liver and stomach (Figure 2A–D). Interestingly, the 8-oxo-dG level was significantly higher in liver tissue than in stomach tissue. Our results showed that the amount of 8-oxo-dG produced by B[a]P was higher in the liver than in the stomach. B[a]P metabolism occurs mainly in the liver, and ROS are produced during B[a]P metabolism, including the quinone-based redox cycle, leading to oxidative DNA damage such as 8-oxo-dG formation [27]. A higher 8-oxo-dG level indicates that B[a]P metabolism actively occurs in the liver, where it produces ROS. Isorhamnetin levels are highest in the liver after quercetin ingestion due to quercetin metabolism. In contrast, quercetin is mainly found in the stomach, because there it remains unmetabolized [28,29]. Therefore, anti-genotoxic effects in the liver and stomach may be attributed to isorhamnetin and quercetin, respectively. To confirm the anti-genotoxic effect of a single compound, quercetin or isorhamnetin was added to HepG2 cells treated with or without B[a]P. Compared with other liver cell lines, the HepG2 liver cancer cell line is most similar to human liver cells; therefore, this line was used for comparing different cellular proteins such as detoxification enzymes [30]. Our results showed that quercetin and isorhamnetin reduced intracellular BPDE-DNA adducts and ROS (Figure 2E,F). 

HepG2 cells were treated with B[a]P for 48 h, after which the amount of intracellular B[a]P was determined (Figure 3A,C). We observed reduced levels of intracellular B[a]P after treatment with quercetin and isorhamnetin, which led us to consider two hypotheses: (1) Quercetin and isorhamnetin prevent the absorption of B[a]P into liver cells, resulting in decreased levels of B[a]P and its metabolites in the cells; and (2) The xenobiotic detoxification process caused an increased metabolic rate, leading to decreased levels of intracellular B[a]P and its metabolites. A previous study found that quercetin treatment did not significantly reduce the B[a]P level of rat bile after B[a]P administration [31]. In addition, our in vitro results showed that the addition of quercetin and isorhamnetin did not change the amount of B[a]P remaining in the cell culture media (Figure 3B,D), which indicates that quercetin and isorhamnetin did not affect the absorption of B[a]P into the cells. Therefore, we further determined the effects of quercetin and isorhamnetin on the xenobiotic metabolism of B[a]P.

Generally, xenobiotic metabolism contributes to the detoxification of poisonous compounds [32]. This process consists of phase I, II, and III, and the enzymes involved in this process are known as xenobiotic-metabolizing enzymes. In the case of B[a]P, phase I enzymes such as CYP1A1 and CYP1B1 oxidize B[a]P to B[a]P-7,8-dihydrodiol and BPDE [33]. In some cases, the intermediates (such as BPDE) produced in phase I can have toxic effects, and these toxic intermediates are stabilized and detoxified in phase II by conjugation with polar compounds such as glucuronides [34]. Moreover, conjugation improves the water solubility of xenobiotic compounds and consequently favors their elimination [35]. Phase II reactions involving BPDE are mediated by transferase enzymes such as GSTA1 and GSTP1 [36]. Finally, conjugated BPDE is pumped out of the cells by phase III enzymes that efflux transporters such as ABCC1 and ABCC2 [30]. In our study, cells treated with either quercetin or isorhamnetin in combination with B[a]P experienced significantly increased gene expression levels of phase I, II, and III enzymes compared with cells that were treated B[a]P only (Figure 4). The protein expression levels of phase I and II enzymes were similar to their gene expression levels (Figure 5). Generally, there are correlations between mRNAs and their protein products due to protein levels being primarily determined by the levels of their transcripts. However, in some cases, the mRNA level by itself is not sufficient to predict protein level, as protein levels are also regulated by posttranscriptional activity. [37]. The ABCC transporter, known as phase III enzymes, is one of the proteins that are significantly affected by posttranscriptional regulation. Especially, micro-RNAs(miRNAs) extensively affect their protein level through interaction with target mRNA [38]. Previous research reported B[a]P induces the mRNA expression of ABCCs, while inhibiting the protein level and function of ABCC transporters [39,40]. In our results, quercetin or isorhamnetin in combination with B[a]P increased both mRNA and protein levels of ABCCs compared to only the B[a]P treated group (Figure 4 and Figure 5). Together, these results indicate that quercetin induces B[a]P oxidation by increasing phase I enzymes, and in turn, the oxidized B[a]P is immediately conjugated by inducing phase II enzymes. Additionally, these results suggest that conjugated BPDE in the cells may be effectively reduced by phase III transporters.

Phase enzyme gene expression is regulated by the transcription factors AhR and NRF2 [41]. The ligand-activated transcription factor AhR activates the expression of CYP genes containing a xenobiotic response element (XRE) in the promoter region [42]. The transcription factor NRF2 induces glutathione S-transferase (GST) and ATP-binding cassette subfamily C (ABCC), which both contain an antioxidant response element (ARE) in the promoter region [43]. Generally, AhR and NRF2 translocate into the nucleus to activate the transcription of their target genes. In addition, NRF2 and AhR have been reported to cross-talk with each other via protein–protein interactions [14]. Therefore, the expression levels of genes involved in xenobiotic metabolism decrease when AhR and NRF2 protein levels are reduced or their translocation is inhibited. Previous studies have suggested that AhR and NRF2 levels affect the level of damage to intracellular DNA [44,45]. In gene knockout models, AhR knockout resulted in increased BPDE-DNA adduct formation compared with the wild type [46], and NRF2 knockout resulted in enhanced oxidative damage compared with the wild type [47]. Our previous study on the attenuation of B[a]P cytotoxicity by silymarin revealed that NRF2 knockdown increases BPDE-DNA adduct formation [48]. Therefore, we hypothesized that (1) quercetin and isorhamnetin increase the expression level of AhR and NRF2, or (2) quercetin and isorhamnetin increase the gene regulatory activity of AhR and NRF2. Our results showed that quercetin and isorhamnetin did not affect the protein levels of AhR or NRF2 (Figure 6A). In addition, we evaluated whether quercetin and isorhamnetin affect the translocation and gene regulatory activities of AhR and NRF2. Our results showed that B[a]P induced translocation of AhR and NRF2; notably, translocation was significantly higher in cells treated with both quercetin or isorhamnetin and B[a]P than in cells treated with B[a]P only (Figure 6B,C). Previous studies have shown that activated AhR and NRF2 translocate to the nucleus, bind to the target promoter, and then induce transcription of the target genes [49,50]. Overall, these results suggest that quercetin induces xenobiotic-metabolizing enzymes by activating AhR and NRF2, thereby reducing B[a]P toxicity. 

It is thought that AhR’s activation by quercetin and isorhamnetin stimulates phase I enzyme transcription, and that the reactive metabolites produced from phase I enzyme activity then stimulate the translocation of NRF2. We confirmed that the increased expression of genes involved in the xenobiotic metabolism of B[a]P can be attributed to the induction of AhR and NRF2 translocation by quercetin and isorhamnetin. 

## 5. Conclusions

Quercetin and its metabolites isorhamnetin and miquelianin are well known for their antioxidant properties. Nevertheless, our results suggest that the reduction of B[a]P-induced cytotoxicity by quercetin and isorhamnetin is not due to antioxidant activity, but rather to other mechanisms. Quercetin and isorhamnetin reduced BPDE-DNA adducts and intracellular B[a]P and its metabolites. The increased gene and protein expression levels of phase I, II, and III enzymes indicated that accelerated xenobiotic detoxification reduced intracellular B[a]P and its metabolites, preventing the formation of BPDE-DNA adducts. Furthermore, quercetin and isorhamnetin induced the translocation of the phase enzyme transcription factors AhR and NRF2. Our results suggest that quercetin and isorhamnetin may exert anti-genotoxic effects by increasing the xenobiotic detoxification metabolism.

## Data Availability

Not applicable.
